# Automatic geometry-based estimation of the locus coeruleus region on T_1_-weighted magnetic resonance images

**DOI:** 10.3389/fnins.2024.1375530

**Published:** 2024-05-07

**Authors:** Iman Aganj, Jocelyn Mora, Bruce Fischl, Jean C. Augustinack

**Affiliations:** ^1^Radiology Department, Athinoula A. Martinos Center for Biomedical Imaging, Massachusetts General Hospital, Boston, MA, United States; ^2^Radiology Department, Harvard Medical School, Boston, MA, United States

**Keywords:** locus coeruleus (LC), image segmentation, magnetic resonance imaging (MRI), expected label value (ELV), U-Net

## Abstract

The locus coeruleus (LC) is a key brain structure implicated in cognitive function and neurodegenerative disease. Automatic segmentation of the LC is a crucial step in quantitative non-invasive analysis of the LC in large MRI cohorts. Most publicly available imaging databases for training automatic LC segmentation models take advantage of specialized contrast-enhancing (e.g., neuromelanin-sensitive) MRI. Segmentation models developed with such image contrasts, however, are not readily applicable to existing datasets with conventional MRI sequences. In this work, we evaluate the feasibility of using non-contrast neuroanatomical information to geometrically approximate the LC region from standard 3-Tesla T_1_-weighted images of 20 subjects from the Human Connectome Project (HCP). We employ this dataset to train and internally/externally evaluate two automatic localization methods, the Expected Label Value and the U-Net. For out-of-sample segmentation, we compare the results with atlas-based segmentation, as well as test the hypothesis that using the *phase* image as input can improve the robustness. We then apply our trained models to a larger subset of HCP, while exploratorily correlating LC imaging variables and structural connectivity with demographic and clinical data. This report provides an evaluation of computational methods estimating neural structure.

## 1 Introduction

The locus coeruleus (LC) is a small elongated hyperpigmented nucleus in the rostral pontine brainstem (Fernandes et al., 2012). It synthesizes most of the brain’s norepinephrine (Aston-Jones and Cohen, 2005) and is involved in various cognitive functions (Sara, 2009). The LC undergoes neuron loss in the early stages of many neurodegenerative diseases (Liu et al., 2017; Betts et al., 2019; Galgani et al., 2020), such as Alzheimer’s disease (Grudzien et al., 2007; Giorgi et al., 2017; Kelly et al., 2017) and Parkinson’s disease (Gesi et al., 2000; Vermeiren and De Deyn, 2017) through the accumulation of tau pathology (Braak et al., 2011; Jacobs et al., 2023) and α-synuclein (Braak et al., 2003), respectively. Non-invasive assessment of the LC integrity *in vivo*, namely via magnetic resonance imaging (MRI), helps to elucidate how LC degeneration relates to the progression and symptoms of neurodegenerative diseases (Liu et al., 2017; Betts et al., 2019; Galgani et al., 2020). Patterns of structural connectivity of the LC to other brain regions—quantified via diffusion MRI (dMRI) (Sun et al., 2020; Levinson et al., 2023)—may further inform us about the pathology distribution in the brain, particularly in the context of Alzheimer’s disease, where some hypothesize that tau protein may transmit neuron to neuron from the LC to other areas (Braak and Del Tredici, 2011).

Quantitative analysis of the LC from MRI requires knowledge of the LC location. Manual annotation of the LC in a large dataset not only necessitates significant expert effort, but also yields a precision limited by moderate inter- and intra-rater variability (Tona et al., 2017). Automatic LC localization (Ariz et al., 2019; Morris et al., 2020; Dünnwald et al., 2021; Sibahi et al., 2023), which is not yet widely available in conventional neuroimaging toolboxes, is therefore highly desirable, as it can facilitate large-scale imaging studies that would have the power to detect subtle changes in the LC in health and disease.

To enhance the contrast of the LC in the MR image, the high concentration of neuromelanin in the LC (Zucca et al., 2006) has been exploited. To that end, several neuromelanin-sensitive MRI sequences (Sasaki et al., 2008) have been successfully employed, including the T₁-weighted (T₁W) Turbo Spin Echo (TSE) (Sasaki et al., 2006; Keren et al., 2009; Tona et al., 2017; Ariz et al., 2019; Dahl et al., 2019) and the magnetization transfer (Nakane et al., 2008; Priovoulos et al., 2018; Liu et al., 2019; Morris et al., 2020; Ye et al., 2021; Jacobs et al., 2023) sequences. The enhanced LC contrast on images acquired with such sequences allows for manual delineation of the LC and the creation of datasets that include gold-standard LC labels. Using a dataset like this for training, a supervised automatic segmentation algorithm can segment the LC on a new similar-contrast image.

Neuromelanin-sensitive MRI, however, typically has a high specific absorption rate (Chen et al., 2014; Liu et al., 2017; Tona et al., 2019), and may also be suboptimal for younger adults due to their lower neuromelanin levels (Zecca et al., 2004; Betts et al., 2019; Liu et al., 2019). Consequently, such a sequence is often not included in large open-access MRI databases of healthy or diseased populations. As for standard MR images (e.g., T₁W images included in almost all MRI databases), the boundaries of the LC cannot be delineated on these images due to the lack of contrast; therefore, the location of the LC can only be approximated at best relative to its surrounding structures using prior neuroanatomical information. Such geometrical localization of the LC might still be useful for some subsequent analyses. For instance, deep neural networks trained on silver-standard labels have shown to be capable of producing more reliable segmentation results than the labels they were trained on (Henschel et al., 2020). Estimating a region that contains the LC may also benefit downstream manual and automatic LC segmentation by providing a region to focus on (the attention mechanism; Xie et al., 2023) and an initial label to refine.

Several LC atlases are publicly available (Keren et al., 2009; Betts et al., 2017; Brooks et al., 2017; Tona et al., 2017; Dahl et al., 2019; Liu et al., 2019; Ye et al., 2021; Levinson et al., 2023), which can be employed to automatize the localization of the LC via atlas alignment and label propagation. The original datasets used to create these public atlases, however, are not generally available. As a result, users cannot apply other supervised segmentation methods to localize the LC, such as those based on modern deep neural networks (Ronneberger et al., 2015). A public database of approximate LC region masks accompanied with corresponding MR images with standard (e.g., T₁W, rather than neuromelanin-sensitive) contrasts is thus desirable. Such a database could help the research community to use existing or new methods to develop automatic LC localization tools that are applicable to many available databases, thereby facilitating large-scale retrospective and prospective analyses involving the LC.

Our contributions in this work are as follows:

- We first manually approximate the LC region on the 3 T T₁W images of 20 subjects of the open-access *Human Connectome Project (HCP)* (Van Essen et al., 2013), sharing the geometrically annotated masks with the research community (see Section 2.4). To our knowledge, other publicly available datasets with manual LC labels instead contain 3T-TSE/7T T₁W images (Tona et al., 2019) or functional MR images (Krebs et al., 2018). Our geometrical estimation is based on dimensional (instead of contrast) information and emphasizes the sensitivity of the detection, thereby resulting in LC masks slightly larger than the LC (i.e., encompassing the LC and some surrounding area).
- We then train two automatic segmentation methods of Expected Label Value (ELV) (Aganj and Fischl, 2021) and U-Net (Ronneberger et al., 2015) on the abovementioned dataset, evaluating the LC localization ability internally as well as on an external dataset (Tona et al., 2019), while also comparing with atlas-based segmentation on the external dataset.
- Inspired by a previous observation (Aganj and Fischl, 2021), we test the hypothesis that using the phase image (i.e., discarding the magnitude Fourier data) would improve the segmentation performance on external datasets (data from different sources).
- We finally apply a trained model to 100 HCP subjects and analyze the volume, image intensity, dMRI measures, and dMRI structural connectivity of the LC masks, correlating them with non-MRI variables.

In the following, we will describe our methods (Section 2), provide our results (Section 3), and discuss them (Section 4).

## 2 Methods

### 2.1 Manual LC region estimation

The human LC is a thin and long column of neurons that extends through multiple levels of the brainstem (Fernandes et al., 2012; Liu et al., 2017). Located in the rostral pons, the LC is on average 14.5 mm long and 2–2.5 mm wide (Fernandes et al., 2012). Due to the lack of LC contrast on T₁W images, we instead used these dimensional landmarks collectively to approximate the LC location: 3 mm lateral from the midline, 1 mm rostral to the fourth ventricle, and 16–20 mm above the pontomedullary junction.

We used Freeview of FreeSurfer (Fischl, 2012) to manually create masks of the areas containing each of the left and right LCs on minimally preprocessed 3T T₁W MPRAGE images (T1w_acpc_dc_restore_brain.nii.gz) of the first 20 subjects of the “100 Unrelated Subjects” group of the HCP (Van Essen et al., 2013) without spatial normalization, which had the volume size of 260 × 311 × 260 with the isotropic voxel size of (0.7 mm)^3^. Our localization approach prioritizes sensitivity to specificity (i.e., includes more voxels than the LC alone), producing masks that are somewhat inflated compared to the actual LC boundaries.

### 2.2 Automatic LC region estimation

#### 2.2.1 Approaches

We trained two supervised image segmentation methods, both implemented in MATLAB, on our 20-subject dataset to automatically approximate the presumptive left or right LC areas (separately), as described below. We binarized the output soft mask and retained the largest connected component.

We used the ELV supervised segmentation (Aganj and Fischl, 2021) (see Section 2.4 for the toolbox) as our first method, which creates a fuzzy map from a combination of labels suggested by all atlas-to-image transformations, weighted by a measure of transformation validity (without explicit deformable registration). The ELV method inherently uses *phase* images as input (obtained by computing the Fourier transform of the image, discarding the magnitude data, and computing the inverse Fourier transform), which we call “ELV (phase).” The map can also be modulated by an image intensity prior (Aganj and Fischl, 2021), i.e., “ELV (phase + image),” to benefit from the image intensity information initially excluded from the phase data.

The second method we used was the U-Net architecture (Ronneberger et al., 2015), which is a convolutional neural network consisting of a contracting path to capture context, a symmetric expanding path for precise localization, and cross connections. We employed a U-Net with two down-sampling layers and 16 initial filters (at the first convolutional layer), with the Dice coefficient as the objective function. We used the Adam optimizer to train the network for 20 epochs on 3D sample patches of size 132 × 132 × 132 with a mini-batch size of 8. We initialized the learning rate as 0.002 and dropped it by 95% every five epochs. The test subject’s LC region was then predicted by averaging the label scores of overlapping patches (stride 10).

#### 2.2.2 Validation

For performance evaluation, we first internally assessed the automatic localization via leave-one-out cross-validation (i.e., trained on 19 images and tested on the remaining image, repeating it for 20 test images). We compared the automatically generated mask with the manual one using the Dice similarity coefficient as the evaluation metric.

For external (out-of-sample) validation, we then applied our models (that had been pretrained on the 20 HCP subjects) to 12 7T T₁W images from the previously unseen dataset by Tona et al. (2019), which had LC labels manually delineated from 3T T₁W TSE images. The images from the latter dataset had the voxel size of 0.70 × 0.64 × 0.64 mm^3^, thereby requiring resampling to match the HCP resolution of (0.7 mm)^3^. Since we trained our models on brain-masked HCP data, we extracted the brain in the new dataset using the SPM12 software package (Ashburner et al., 2014) (it failed for one subject, which we excluded).

Next, we used our external validation setup to test the performance of the commonly used atlas-based segmentation approach. We used the image-to-MNI deformation fields provided in the HCP dataset to propagate the images and our LC masks of the 20 HCP subjects to the MNI space, which we then averaged to create an MNI atlas with fuzzy masks. We then ran our implementation (Aganj et al., 2017) of diffeomorphic-demons atlas-to-image (asymmetric) deformable registration (Vercauteren et al., 2009) to align the intensity-normalized MNI atlas to each of the external-validation (Tona et al., 2019) intensity-normalized images and transformed the LC masks from the atlas to the test image space and binarized them (see Section 2.4 for the toolbox). We did so after heuristically fine-tuning the regularization and step-size parameters of registration on a few subjects.

Note that our geometrical LC approximation produces a generous area containing the LC, and, as such, comparing it to the specific label of the actual LC in external validation results in a suboptimal Dice score. For instance, if our localized area has a volume α times larger than that of the LC label, then the Dice will be no higher than $2/\left(1+\alpha \right)$ . Nonetheless, this comparison can still help to assess how much our automatic inflated LC neighborhood overlaps with the LC.

#### 2.2.3 Phase image as input

We have previously observed that ELV (phase + image) outperformed ELV (phase) in internal cross-validation, but not in external out-of-sample validation (Aganj and Fischl, 2021). The phase image—constructed by taking the Fourier transform, dividing by the magnitude Fourier, and taking the inverse Fourier transform—remains real-valued with voxels that are to some extent still spatially correlated. Instead of the original image intensity values, however, the phase image contains enhanced edges and region borders. We hypothesized that, being less sensitive to inter-dataset variation in image intensity, the phase image might be more robust to domain shift, resulting in better model performance than the image itself would in external validation. We tested this by comparing the two abovementioned ELV variations, as well as comparing the original U-Net [“U-Net (image)”] to a variation of it that received the phase image as input [“U-Net (phase)”] and a two-channel-input variation that received both the image and the phase as input [“U-Net (image + phase)”]. For robustness of the phase image, we divided the Fourier transform by its magnitude *plus* a constant (0.001 times the norm of the Fourier transform).

Prompted by the different image intensity distributions of our two datasets, we also experimented with normalizing the input image by its intensity standard deviation during both training and testing (denoting the normalized image with “nmz”), which led to additional model variations such as “ELV (phase + nmz),” “U-Net (nmz),” and “U-Net (nmz + phase).” For the latter two U-Net variations, the input layer also performs patch-wise zero-meaning and normalization.

### 2.3 Analysis of HCP data

We trained the default implementation of the U-Net on our 20 HCP subjects and then applied it to the “100 Unrelated Subjects” group of the HCP. We used the resulting left and right LC masks to compute their volumes as well as the mean T₁W and T₂W image intensities inside them. We also propagated the soft masks to the (1.25 mm)^3^ isotropic-voxel dMRI space (using HCP’s T1w_acpc_dc_restore_1.25.nii.gz image as target), before binarizing them in the dMRI space. We then similarly computed the mean fractional anisotropy (FA) and the mean apparent diffusion coefficient (ADC), resulting in a total of 10 LC (regional) imaging variables per subject.

Next, we performed an exploratory analysis to correlate the imaging variables with 504 non-MRI variables (demographics, medical history, family history, dementia/cognitive exam scores, personality/emotion tests, motor/sensory tests, task performance, etc.). We Bonferroni-corrected the Pearson’s correlation *p*-values for multiple comparisons through multiplication by the numbers of imaging and non-MRI variables, i.e., *p_B_* = *p* × 10 × 504. We visually inspected relationships with *p_B_* < 0.05 to exclude any spurious correlations due to outliers (e.g., avoiding situations with most data points clustered together with no obvious relationship), reporting the surviving significant correlations.

Finally, we quantified dMRI-derived structural connectivity of the LC area to the rest of the brain, and examined the associations between the connectivity pattern of LC and non-MRI variables. We used our open-source toolbox (see Section 2.4) to reconstruct the diffusion orientation distribution function in constant solid angle (Aganj et al., 2010), perform Hough-transform global probabilistic tractography (Aganj et al., 2011), compute a symmetric structural connectivity matrix between a set of brain regions including the two computed LC areas and other regions segmented by FreeSurfer (Fischl, 2012), and augment the raw matrices with indirect connections (Aganj et al., 2014). For each subject, we generated a 2 × 86 matrix representing the connectivity strength between each LC area and 86 other brain regions (contralateral LC + 85 FreeSurfer-generated regions). We then correlated each element of this matrix with non-MRI variables across the population, while Bonferroni-correcting the *p*-values for both the number of connections and the number of variables. We have previously described this pipeline in detail (Aganj et al., 2023).

### 2.4 Data and toolboxes

Our manually annotated (enlarged) LC masks are publicly available at: https://www.nitrc.org/projects/lc20.

Magnetic resonance images were provided by:

- The Human Connectome Project (HCP, RRID:SCR_006942) (Van Essen et al., 2013), WU-Minn Consortium (Principal Investigators: David Van Essen and Kamil Ugurbil): https://www.humanconnectome.org/study/hcp-young-adult.
- Klodiana-Daphne Tona et al. (2019): https://doi.org/10.34894/PMQHZD.

The following toolboxes were used for data processing and analysis:

- Our MATLAB (RRID:SCR_001622) toolboxes for:

- The expected label value (ELV) supervised image segmentation (Aganj and Fischl, 2021): https://www.nitrc.org/projects/elv.
- The reconstruction of the orientation distribution function in constant solid angle (Aganj et al., 2010), Hough-transform tractography (Aganj et al., 2011), and connectivity matrix computation and augmentation (Aganj et al., 2014): http://www.nitrc.org/projects/csaodf-hough.
- Mid-space-independent deformable image registration (Aganj et al., 2017): https://www.nitrc.org/projects/msi-register.

- FreeSurfer (RRID:SCR_001847) (Fischl, 2012): https://freesurfer.net.
- SPM12 (RRID:SCR_007037) (Ashburner et al., 2014): https://www.fil.ion.ucl.ac.uk/spm/software/spm12.

## 3 Results

### 3.1 Manual LC region estimation

**Figure 1:**
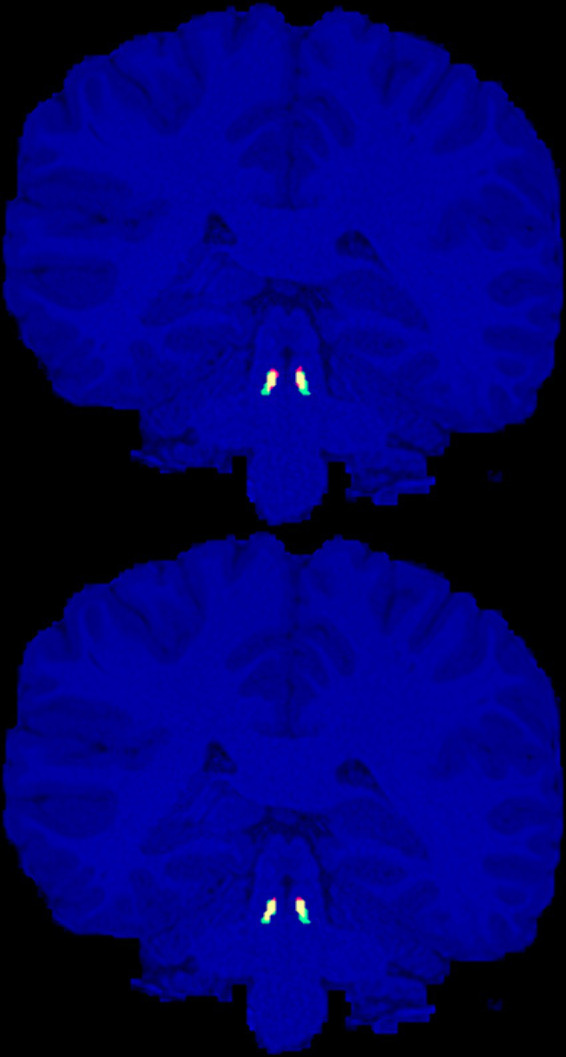
Manually annotated presumptive LC areas (green), automatic localization of LC using the ELV (top) and U-Net (bottom) methods (red), and their overlap (yellow), are shown on the coronal slice with the largest intersection with the manual LC areas for the representative HCP subject (with the median ELV Dice score).

We have provided our geometrically annotated (enlarged) LC masks for the 20 HCP subjects to the public (Section 2.4). As expected, we did not observe any LC contrast on T₁W images to guide the manual delineation of the LC region. Figure 1 shows the approximated LC masks (in green + yellow) for a representative subject.

The volume of the left and right LC regions had a cross-subject mean ± standard error of the mean (SEM) of 19.5 ± 0.8 and 19.8 ± 0.8 mm^3^, respectively. We also measured this for the manual labels in the external (Tona et al., 2019) dataset; the cross-subject average volumes of the left and right LC labels were 6.9 ± 0.7 and 7.4 ± 0.8 mm^3^, respectively. Two-sided paired *t*-tests between the left and right volumes did not reveal a statistically significant difference between them in either dataset.

### 3.2 Automatic LC region estimation

We assessed our variations of the ELV and the U-Net methods (Section 2.2) via leave-one-out cross-validation on the 20-subject subset of the HCP as well as on the external dataset by Tona et al. (2019). Table 1 summarizes the median, mean, and SEM of the Dice scores.

**Table 1:** Median, mean, and standard error of the mean (SEM) of the Dice score, measuring the overlap of the automatic and manual LC regions.

Method	Input	Cross-validation Dice score		External validation Dice score	
		*(median)*		*(median)*	
		*(mean ± SEM)*		*(mean ± SEM)*	
		Left LC	Right LC	Left LC	Right LC
ELV	Phase	0.62	**0.644**	0.04	0.11
		0.61 ± 0.01	**0.64** ± 0.02	0.09 ± 0.04	0.11 ± 0.03
	Phase + image	0.63	0.643	0.04	0.11
		0.62 ± 0.02	0.63 ± 0.02	0.09 ± 0.04	0.11 ± 0.03
	Phase + nmz	0.61	0.636	0.04	0.11
		0.61 ± 0.02	0.63 ± 0.02	0.10 ± 0.04	0.12 ± 0.03
U-Net	Image	0.65	0.61	0.00	0.00
		0.61 ± 0.03	0.60 ± 0.02	0.00 ± 0.00	0.02 ± 0.02
	nmz	**0.670**	0.63	0.22	0.20
		0.63 ± 0.02	0.60 ± 0.02	0.20 ± 0.02	0.20 ± 0.02
	Phase	0.64	0.636	0.00	0.22
		0.62 ± 0.03	0.61 ± 0.02	0.07 ± 0.05	0.26 ± 0.03
	Image + phase	0.63	0.60	0.00	0.00
		**0.64** ± 0.02	0.58 ± 0.03	0.00 ± 0.00	0.00 ± 0.00
	nmz + phase	0.62	0.63	0.13	0.29
		0.59 ± 0.03	0.62 ± 0.02	0.16 ± 0.02	**0.29** ± 0.02
Atlas-based	nmz	-		**0.27**	**0.35**
				**0.21** ± 0.06	0.25 ± 0.06

The highest value in each column is shown in bold.

By combining the left and right LC results together, the highest cross-validation Dice scores were achieved by ELV (phase) in terms of the mean (0.62 ± 0.01) and by U-Net (nmz) in terms of the median (0.65). Figure 1 shows the labels generated by both methods for the representative subject with median ELV Dice score. We then combined the best performing ELV (phase) and U-Net (nmz) results by multiplying their soft masks and binarizing them, leading to median (mean) Dice scores of 0.667 (0.66 ± 0.02) and 0.66 (0.64 ± 0.02) for the left and right LC, respectively, showing a slight improvement compared to individual ELV and U-Net results.

The external validation Dice scores were considerably lower, with the best left + right mean (0.22 ± 0.02) and median (0.22) scores obtained by U-Net (nmz + phase). The median and mean sensitivity for this method were 0.63 and 0.63 ± 0.03, respectively. Image normalization (nmz) improved the external validation Dice in all cases.

The results of the atlas-based approach, compared to the U-Net, showed overall improvement for the left LC, and increased median but decreased mean Dice score for the right LC (Table 1). The latter was mainly due to the subpar performance of deformable registration for 4 out of 11 subjects (whereas such registration was not required for the U-Net and ELV methods). The median and mean sensitivity of the atlas-based approach, 0.43 and 0.36 ± 0.06, were, however, lower than those of the U-Net.

Left and right LC Dice scores were significantly correlated with each other in most cross-validation results by both methods [ELV (phase): *r* = 0.58, *p* = 0.007; U-Net (image): *r* = 0.61, *p* = 0.004] and in the external validation results by the ELV [(phase + nmz): *r* = 0.89, *p* = 0.0003] and the atlas-based (*r* = 0.85, *p* = 0.001) approaches.

### 3.3 Findings from HCP

**Figure 2:**
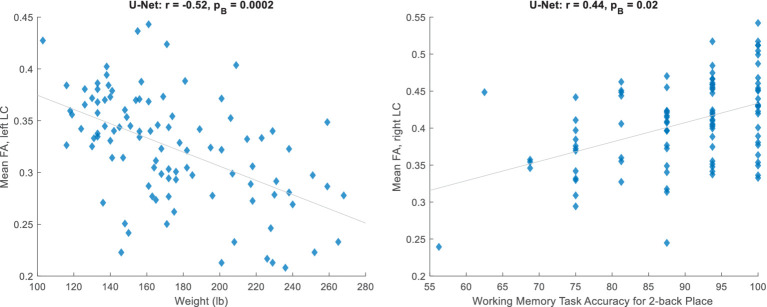
Significant relationships between the mean fractional anisotropy (FA) inside the automatically estimated LC region and non-MRI variables (corresponding to Table 2, unadjusted for ICV).

Next, we trained a U-Net (with the default implementation, which receives the image and normalizes the patches at its first layer) on the 20 subjects and applied it to 100 HCP subjects. After correlating non-MRI variables with imaging variables (Section 2.3), the only correlations surviving the Bonferroni correction were some with the mean FA, all of which passed the visual inspection. Table 2 lists these significant relationships, mainly with the body weight and memory, and Figure 2 shows two examples. Adjusting for the intracranial volume (ICV) improved the correlation significance with the memory task accuracy, but reduced that with the body weight.

**Table 2:** Pearson’s correlation coefficients (*r*) and Bonferroni-corrected *p*-values (*p_B_*) of the significant correlations of the mean fractional anisotropy (FA) inside the computed LC area with non-MRI variables, without and with intracranial volume (ICV) adjustment.

Non-MRI variable	No ICV adjustment		Adjusted for ICV	
	Left LC	Right LC	Left LC	Right LC
Body weight	*r* = −0.52	*r* = −0.52	*r* = −0.44	*r* = −0.44
	*p*_B_ = 0.0002	*p*_B_ = 0.0002	*p*_B_ = 0.03	*p*_B_ = 0.02
Body mass index (BMI)	-	*r* = −0.44	-	*r* = −0.43
		*p*_B_ = 0.02		*p*_B_ = 0.04
Body mass index (BMI) at heaviest time	-	*r* = −0.43	-	*r* = −0.43
		*p*_B_ = 0.04		*p*_B_ = 0.046
Working memory task accuracy: two-back place	-	*r* = 0.44	-	*r* = 0.46
		*p*_B_ = 0.02		*p*_B_ = 0.009

We lastly computed the strength of connectivity between each LC area and 86 other brain regions (85 + contralateral LC). After Bonferroni correction for all possible structural connections to the LC and for all non-MRI variables, none of the few significant correlations between the two passed the visual inspection (see Section 2.3).

## 4 Discussion

We have presented a new dataset of high-resolution (isotropic 0.7 mm voxel) LC areas manually approximated for 20 subjects of the HCP. This dataset is publicly available to the research community (Section 2.4), allowing the development of supervised tools applicable to standard 3T T₁W MPRAGE (rather than neuromelanin-sensitive) MRI for quantitative analysis using large existing or future MRI databases. Given the lack of LC contrast on standard T₁W images, we used dimensional information instead to estimate the region that approximately included the LC. We emphasized sensitivity for this task and created masks that were slightly larger than and contained the LC (which, if desired, could be shrunk in post-processing via the *erosion* operation). The masks had a bilateral mean volume of 19.7 ± 0.6 mm^3^, expectedly larger than the LC volume reported in the literature, such as 6.6 mm^3^ (Ariz et al., 2019), 7.2 mm^3^ (that we computed from the dataset) (Tona et al., 2019), 9.5 mm^3^ (Tona et al., 2017), 12.8 mm^3^ (Theofilas et al., 2017), and 16.7 mm^3^ (Schwarz et al., 2017).

Our internal evaluation of (the optimal variations of) the ELV and U-Net automatic segmentation approaches on our data resulted in cross-validation Dice scores with the mean of 0.62 (by ELV) and median of 0.65 (by U-Net). In comparison, the LC label Dice scores reported in the literature for inter-rater reliability are 0.50 (Ariz et al., 2019), 0.54–0.64 (Tona et al., 2017), 0.64 (Tona et al., 2019), and 0.68 (Dünnwald et al., 2021), for scan-rescan reliability are 0.24–0.48 (Sibahi et al., 2023) and 0.63 (Langley et al., 2017), and for automatic segmentation are 0.40 (Ariz et al., 2019), 0.54–0.64 (Sibahi et al., 2023), and 0.60–0.71 (Dünnwald et al., 2021).

Our external validation resulted in lower mean bilateral Dice scores (ELV: 0.11, U-Net: 0.22, atlas-based: 0.23). Several reasons could account for this. Given that the average volume of our presumptive LC areas was α = 2.7 times larger than that of the LC labels in the test (Tona et al., 2019) dataset, the Dice score between the two was capped at 0.54 (see Section 2.2.2). The cap was possibly even lower due to inter-rater variability between the two datasets, especially since our LC areas were based on dimensional information whereas the labels in the external dataset were delineated based on the LC contrast seen on neuromelanin-sensitive (3T T₁W TSE) images. Another factor contributing to the lower Dice value may have been the domain shift, particularly caused by the different MRI field strengths of the input training (3 T) and test (7 T) images, which are known to reveal different MRI tissue properties (van der Zwaag et al., 2016).

Our comparative experiment with the popular atlas-based approach produced higher accuracy conditioned to the success of atlas-to-image deformable registration, which was not always the case. While we made a reasonable effort to fine-tune the registration parameters, the optimal parameter values might depend on the image (as opposed to being fixed for the dataset), possibly making the application of this approach more cumbersome than other approaches (e.g., ELV and U-Net) that do not require deformable registration.

We tested the hypothesis that using phase images could enhance out-of-sample segmentation. The U-Net indeed achieved the highest bilateral external-validation accuracy when the input included the phase image. The different field strengths and acquisition protocols of the training (HCP) and test (Tona et al., 2019) datasets may have caused inconsistencies in their image intensities. The phase image improved our external validation results by ignoring the Fourier-domain magnitude information, which possibly alleviated such inter-database inconsistencies to some extent.

The Dice scores corresponding to the approximated left and right LC regions were often significantly correlated with each other, perhaps due to their correlation with the image quality and variance of the test subject.

Following image segmentation of 100 unrelated HCP subjects and an exploratory analysis, we found significant correlations with the mean FA inside the LC region, mainly negative correlations with the body weight and a positive correlation with working memory. These correlations might also be partially driven by other neighboring small nuclei and white matter, given that our estimated region is larger than the LC itself and there may also be residual misalignment between the T₁W and dMRI images. In a similar analysis, LC connectivity was found not to be significantly correlated with non-MRI variables, possibly due to the homogeneity and narrow age range (22–36 years old) of the healthy HCP cohort (Aganj et al., 2023). Our stringent Bonferroni correction for all compared variables and LC connections may additionally have led to type II errors (false negatives). In most related HCP studies, the LC connectivity has been measured to predefined ipsilateral target regions pertinent to disease, such as the transentorhinal cortex (Sun et al., 2020) and limbic regions (Levinson et al., 2023). In contrast, we ran whole-brain tractography to explore all regions’ potential connectivity to the LC, which is especially important considering the LC’s extensive axonal branching innervating diverse remote areas throughout the brain (Sara, 2009).

This report has the following limitations, because of which we do not imply our LC masks to be anatomically accurate LC labels and caution their use where specific and accurate labels are required.

- The LC masks were not based on anatomical contrast and the location is approximate. We were not able to directly compare our masks with those derived from neuromelanin-sensitive images, given that the HCP database did not include such contrasts. The aim of this study was to assess the applicability of the T₁W contrast, which is available to most researchers, for LC localization. Future research will need to evaluate the compatibility of such LC masks with those drawn on neuromelanin-sensitive (e.g., TSE) images of the same subjects.
- Given its small size, the LC is difficult to model and partial voluming effects can introduce noticeable error in the imaging quantity derived from it. Should a non-MRI variable and the error in an imaging variable happen to be related to each other (e.g., by both being correlated to a third factor such as the ICV), a spurious relationship might appear in the correlation analysis. Thorough investigation of the automatically misclassified voxels to identify any potential bias is a subject of our ongoing research.
- Supervised learning methods often perform better with more training data. Although we created a modest-sized training set of 20 subjects (40 bilateral masks), image segmentation (especially with deep neural networks) has shown promise in learning from even small datasets (Lee et al., 2019), possibly due to each voxel in the image serving as a data point.

Quantitative MRI studies of the LC—requiring automatic LC region estimation—have the potential to generate imaging biomarkers for early diagnosis of neurodegenerative diseases and patient stratification (Betts et al., 2019). Making our LC neighborhood localization algorithm more specific and integrating it into FreeSurfer (Fischl, 2012) are subjects of our future work.

## Data availability statement

The original contributions presented in the study are included in the article (see Section 2.4); further inquiries can be directed to the corresponding author.

## Ethics statement

Ethical approval was not required for the study involving humans in accordance with the local legislation and institutional requirements. Written informed consent to participate in this study was not required from the participants or the participants’ legal guardians/next of kin in accordance with the national legislation and the institutional requirements.

## Author contributions

IA: Conceptualization, Data curation, Formal analysis, Funding acquisition, Investigation, Methodology, Project administration, Software, Supervision, Validation, Visualization, Writing – original draft, Writing – review & editing. JM: Data curation, Resources, Writing – review & editing. BF: Conceptualization, Methodology, Writing – review & editing. JA: Conceptualization, Data curation, Investigation, Methodology, Resources, Writing – review & editing.
